# Cx43 deficiency confers EMT-mediated tamoxifen resistance to breast cancer via c-Src/PI3K/Akt pathway

**DOI:** 10.7150/ijbs.55453

**Published:** 2021-06-11

**Authors:** Deng-Pan Wu, Yan Zhou, Li-Xiang Hou, Xiao-Xiao Zhu, Wen Yi, Si-Man Yang, Tian-Yu Lin, Jin-Lan Huang, Bei Zhang, Xiao-Xing Yin

**Affiliations:** 1 Jiangsu Key Laboratory of New Drug Research and Clinical Pharmacy, Pharmacy School of Xuzhou Medical University, Xuzhou City, Jiangsu Province, 221004, P.R. China.; 2 Department of Pharmacology, Pharmacy School of Xuzhou Medical University, 221004, Xuzhou City, Jiangsu Province, P.R. China.; 3 Scientific research center of traditional Chinese medicine, Guangxi University of Chinese Medicine, Nanning City, Guangxi Zhuang Autonomous Region, P.R. China.; 4 Clinical Pharmacy, Jingjiang People's Hospital, 214500, Jingjiang City, Jiangsu Province, P.R. China.; 5 Department of gynaecology and obstetrics, Xuzhou Central Hospital, 221009, Xuzhou City, Jiangsu Province, P.R. China.

**Keywords:** Connexin 43, tamoxifen resistance, epithelial-mesenchymal transition, c-Src/PI3K/Akt pathway

## Abstract

Tamoxifen (TAM) resistance has indicated a significant challenge during endocrine therapy for hormone-sensitive breast cancer. Thus, it is significant to elucidate the molecular events endowing TAM resistance to endocrine therapy. In this study, we found that epithelial-mesenchymal transition (EMT) was an important event to confer TAM resistance, and attenuating EMT by elevating connexin (Cx) 43 expression could reverse TAM resistance. Specifically, Cx43 overexpression improved TAM sensitivity, while Cx43 depletion facilitated TAM insensitivity by modulating EMT in T47D TAM-resistant and -sensitive cells, and transplanted xenografts. Importantly, we found a novel reciprocal regulation between Cx43 and c-Src/PI3K/Akt pathway contributing to EMT and TAM resistance in breast cancer. Moreover, we identified that Cx43 deficiency was significantly correlated with poor relapse-free survival in patients undergoing TAM treatment. Therefore, Cx43 represents a prognostic marker and an attractive target for breast cancer treatments. Therapeutic strategies designed to increase or maintain Cx43 function may be beneficial to overcome TAM resistance.

## Introduction

Breast cancer is one of the most common cancer and the second leading causes of cancer-related deaths in women worldwide. Approximately 70%-75% of breast cancer are classified as estrogen receptor-positive (ER^+^) breast cancer 1. For ER^+^ breast cancer, primary therapy is endocrine treatment. There are reports showing that antiestrogen treatment can dramatically improve the treatment outcome and reduce the recurrence risk for patients with ER^+^ breast cancer 2. Tamoxifen (TAM) is the most widely used nonsteroidal and antiestrogen agent for ERα^+^ patients 3. Although initial responses to TAM treatment are positive, approximately 30% of ER^+^ patients have acquired resistance to TAM therapy and eventually develop local recurrence and distant metastases 1. Therefore, exploring the molecular mechanisms of TAM resistance and investigating novel targets to overcome acquired TAM resistance in breast cancer is urgently needed.

Epithelial-mesenchymal transition (EMT) is a process by which epithelial cells lose their junctions and polarity and gain migratory and invasive phenotypes to transform into mesenchymal stem cells. The features of EMT include changes in cellular morphology, downregulation of the expression of epithelial cell markers such as E-cadherine (E-ca) and upregulation of mesenchymal markers such as N-cadherin (N-ca) and α-SMA 4. Emerging data highlight that EMT in cancer cells is associated with increased tolerance to chemotherapy and radiotherapy 5, while reversing EMT in resistant cancer cells could increase the cytotoxicity of cancer therapeutic agents 6, 7. Consistent with these findings, EMT features, including increased cell motility and morphological distinctions have been observed in TAM-resistant human breast cancer cells 8. Inducing EMT phenotype by TGF-β1 or MCAM/CD146 promotes TAM resistance in breast cancer cells 9, 10. Thus, reversing EMT has been considered as a beneficial strategy to conquer acquired TAM resistance in breast cancer.

Connexins (Cxs) are a family of transmembrane proteins, which compose gap junction (GJ) channel between neighboring cells. Gap junction intercellular communication (GJIC) is driven by the diffusion of the signaling molecules smaller than 2kDa 11. It is generally believed that the tumorigenic phenotype is widely associated with lack of Cx expression and GJIC, while restoring Cx expression and GJIC improved the sensitivity of cancer cells to chemotherapeutics and inhibited tumor growth 12. Cx43 is the most widely studied isoform of Cxs due to its abundant expression in the heart, breast, brain and colorectal tissues 13. It has been proven that Cx43 and its derived GJIC act as tumor suppressors to inhibit glioma and colorectal cancer growth 14, 15. Additionally, Cx43 sensitizes malignant cells to platinum-based drugs. For example, increasing the level of Cx43 could improve oxaliplatin cytotoxicity in colorectal cancer cell lines 16 and enhance the sensitivity of glioma to temozolomide 14. Additionally, improving Cx43-mediated gap junctional function by α-connexin carboxyl-terminal (ACT1) peptide could increase the activity of lapatinib and TAM 17. Nevertheless, the underlying mechanisms of Cx43 in TAM resistance in breast cancer remain elusive.

In this study, we have explored the potential role of Cx43 in TAM resistance in breast cancer and its underlying mechanisms. We demonstrated that Cx43 deficiency conferred TAM resistance and overexpression of Cx43 reversed TAM resistance by modulating EMT. Importantly, we found that there was a mutual regulation between Cx43 and c-Src/PI3K/Akt pathway contributing to EMT and TAM resistance. Additionally, by analyzing the association of Cx43 expression with the outcome of TAM treatment, we observed that Cx43 deficiency predicts poor clinical outcome to TAM therapy. This study indicates that Cx43 could serve as a critical regulator in TAM resistance. Therapeutic strategies designed to elevate or sustain Cx43 function may be beneficial to overcome TAM resistance.

## Methods

### Materials

TAM and 18α-GA were provided by Sigma-Aldrich (St. Louis, MO, USA). TGF-β1 was obtained from Pepro Tech (Rocky Hill, NJ, USA). MS-275 was supplied by MedChemExpress (Monmouth Junction, NJ, USA). Antibodies against Cx43 and α-SMA were obtained from Abcam (Cambridge Science Park, Cambridge, England). E-ca, N-ca, p-Akt, Akt, c-Src and p-c-Src antibodies and LY294002 (PI3K inhibitor) were purchased from Cell Signaling Technology (Danvers, MA, USA). β-actin and GAPDH antibodies were purchased from Bioworld Technology, Co, Ltd. (Nanjing, China). Dasatinib was from Aladdin Bio-Chem Technology Co.LTD (Shanghai, China). IGF-1 was from PeproTech, Inc. (Rocky Hill, USA). Other reagents were all provided from Sigma-Aldrich unless otherwise noted.

### Cell culture and treatment

Mycoplasma-free T47D cells were obtained from the American Type Culture Collection (Manassas, VA, USA). Cells were cultured in RPMI1640 (Gibco, Grand Island, NY, USA) containing 10% fetal bovine serum (Gibco, Grand Island, NY, USA) and 1% penicillin/streptomycin at 37 °C in water-saturated 5% CO_2_ atmosphere. TAM-sensitive T47D (T47D/TS) cells were exposed to increasing concentrations of TAM and maintained in 10^-6^ M TAM for at least 18 months to establish acquired TAM-resistant T47D (T47D/TR) cells. Cells were incubated with 25μmol/L LY294002 or 2 μmol/L dasatinib for 24 h to retard the activity of PI3K/Akt signaling or c-Src, respectively. Additionally, cells were treated with IGF-1 (100 ng/mL) for the activation of PI3K/Akt signaling.

### Cx43 interference and overexpression

The lentiviral pGLV3/H1/GFP+Puro vector (GenePharma, China) containing shRNA against Cx43 and pCDH/CMV/MCS/EF1/coRFP+Puro vector (GenePharma, China) containing chimeric Cx43 was constructed as previously reported 18. shRNA with no target gene (scramble) was considered as a control. Lentivirus stably transfected to T47D/TS and T47D/TR cells in the presence of 5µg/mL polybrene (GenePharma, China). After 2 weeks, single independent clones were randomly isolated and plated separately to analyze for Cx43 knockdown or overexpression by western blotting.

### Western blotting

Western blotting protocol was according to previous studies 19, 20. Briefly, the extracted protein samples were separated and transferred to a nitrocellulose transfer membrane (Excell Bio, China). Primary antibodies (dilution ranging from 1:2000 to 1:5000) were incubated at 4 °C overnight. Secondary antibody was IRDye 800CW purified immunoglobulin-conjugated anti-rabbit (dilution 1:10000). Immunopositive bands were visualized at Ex/Em=778nm/795nm.

### CCK-8 assay

TAM cytotoxicity was assessed by a CCK-8 assay (Dojindo Molecular Technologies, Japan). As previously reported 21, after incubation with different concentrations of TAM for 48 h, 10 μL CCK-8 solution was added and incubated with cells for 2 h. The OD values were detected at 450 nm to assess cellular survival using Enzyme-labeling instrument (Elx808, Bio Tek, America).

### “Parachute” dye-coupling assay

GJIC function was evaluated by “Parachute” dye-coupling assay was performed as described in our previous study 22. Donor and receiver cells were grown to confluence. Donor cells were loaded with 5 µM calcein-acetoxymethyl ester, which is converted into the gap junction-permeable dye calcein and diffuse through GJ channel. The donor cells were then seeded onto the receiver cells at 1:150 donor/receiver ratio after trypsinization. The donor cells were allowed to contact with the monolayer of receiver cells for GJ formation for 4 h at 37 °C. The average amount of the receiver cells receiving dyes per donor cell was determined using a fluorescence microscope and normalized to the controls.

### Cell invasion assay

Cell invasion assay was performed to assess the invasive ability as described in a previous study 23. Briefly, the invaded cells were counted in eight high-power fields of cells in each well under an inverted microscope. The relative invasive rate was determined by the invasive rate of the treated group (number of invaded cells per total cell number) divided by that of the control group.

### *In vivo* xenograft studies

BALB⁄c-nude mice (16-22 g, female) were subcutaneously inoculated under the right shoulder with 5 ×10^7^ cells/flank. One week before injection, the mice transplanted with Cx43-deficient and its scramble T47D/TS cells were pretreated with drinking water containing 1 μM β-estradiol (Sigma-Aldrich). The mice transplanted with Cx43-overexpressed and control T47D/TR cells were provided with drinking water supplemented with 1 μM β-estradiol (Sigma-Aldrich) and pasted with estradiol sustained-release patch (1.25 mg per mice) (Zhejiang Yatai Pharmaceutical Co., Ltd., Zhejiang, China). When the volume of tumors reached to 100 mm^3^, the mice were intragastrically administrated with vehicle (control) or 30 mg/kg TAM for 10 days. Tumor dimensions were measured by a caliper every 2 days. The tumor volume (V) was calculated as (length×width^2^) × 0.5. The relative tumor volume (RTV) of each tumor was defined as the ratio of the volume at a given time and the volume prior to treatment 24. On day 10, after their tumor sizes were measured, all mice were sacrificed and the xenografts were excised and weighted. Meanwhile, the expression of EMT biomarkers of xenografts were detected. All animal experiments were approved by the Guidance Suggestions for Caring for Laboratory Animals issued by the Ministry of Science and Technology of China in 2006.

### Immunohistochemistry (IHC) for tissue microarray

Commercial breast cancer tissue microarray (TMA) slides (BC081120e and HBreD140Su05) were purchased from US Biomax, Inc. (Maryland, USA) and Shanghai Outdo Biotech Co. Ltd. (Shanghai, China), respectively. All tissues were collected with the patients consenting to the use of their tissues and clinical data. IHC staining against Cx43, E-ca and α-SMA was carried out with EliVision^TM^plus kit (Maxim biotechnology co. Ltd, Fuzhou, China). The staining was evaluated blindly and independently by two pathologists. The intensity of staining was scored 0 to 3 (0=negative, 1= low, 2=moderate, 3=high). The percentage of positively stained cells was recorded and divided into four categories: 1 (0%-25%), 2 (26%-50%), 3 (51%-75%) and 4 (76%-100%). The expression level was calculated by multiplying the scores of staining intensity and the percentage of positive cells 25.

### Co-IP assay

Cell samples were lysed in iced cold PIPA buffer and the supernatants were collected by centrifuging at 14000 rpm. The lysates were incubated with Cx43 antibody and protein A/G agarose gel overnight. The gel was washed with RIPA buffer, boiled with SDS loading buffer and subjected to SDS-PAGE resolution. After SDS-PAGE, c-Src antibody was used to detect the interaction of these two proteins by Western blotting assay 26.

### Statistical analysis

The data were expressed as mean±SD. The variance was analyzed using the SPSS software for Windows 17.0 using one-way analysis of variance (ANOVA) or *t*-test. *P*<0.05 was considered to be statistically significant.

## Results

### Cx43 expression significantly correlates with clinicopathological parameters and clinical outcome to TAM therapy

**Figure 1:**
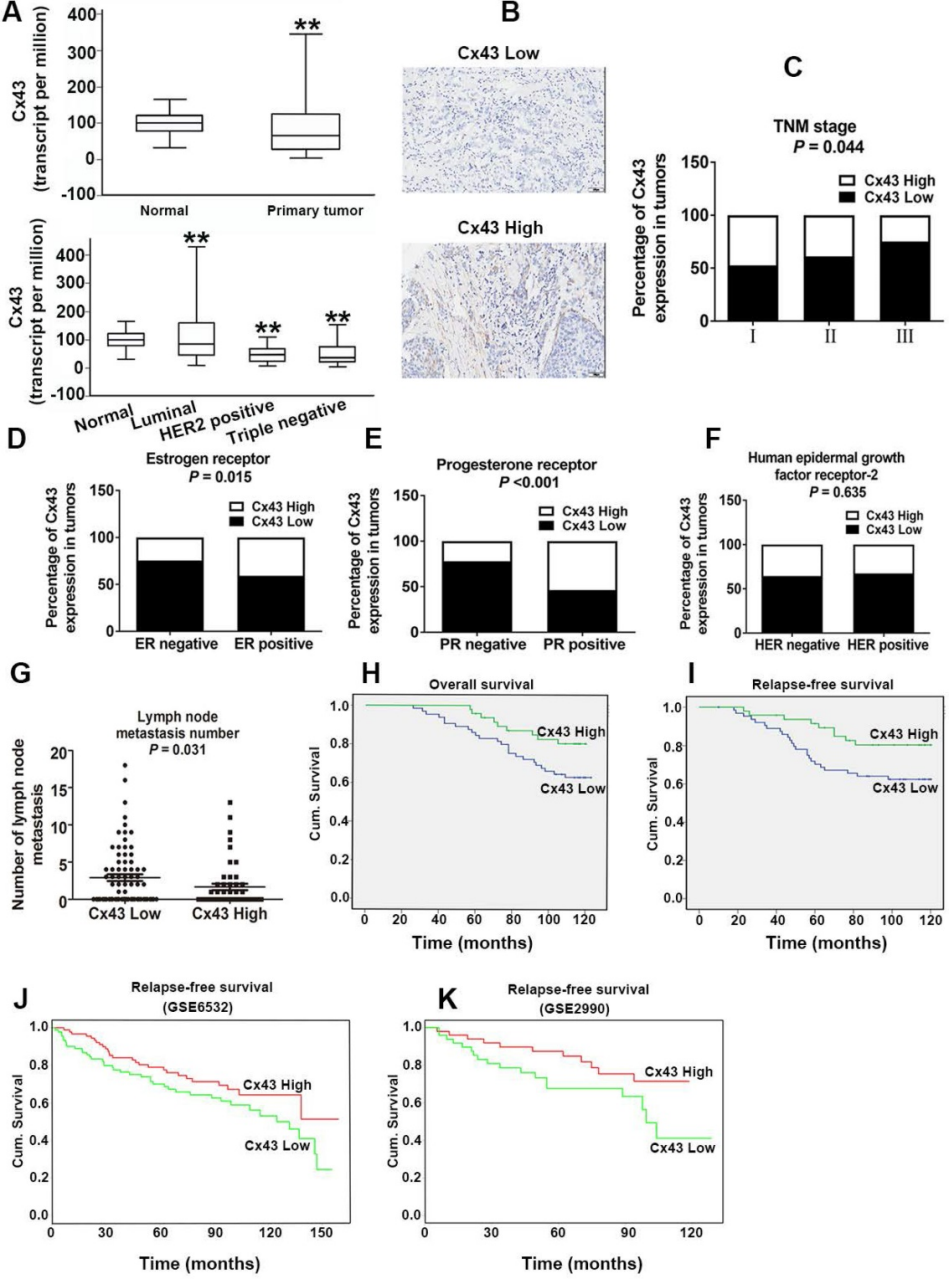
Cx43 expression significantly correlates with clinicopathological parameters and clinical outcomes of TAM therapy. (A) Cx43 mRNA expression in primary breast cancer and its subtypes from TCGA database. **P<0.01 *versus* normal breast tissue. (B) Weak and strong Cx43 staining in breast cancer tissue. Scale bar, 100 μm. (C-F) Percentages of human breast cancer samples with high level of Cx43 expression in different tumor subtypes and tumor grades (χ^2^ test). (G) Correlation of Cx43 expression with lymph node metastasis number (unpaired *t*-test). (H, I) High Cx43 expression correlated with a higher 10-year overall survival and relapse-free survival for 127 breast cancer patients (*P*<0.05, log-rank test). (J, K) High Cx43 expression correlated with a higher 10-year relapse-free survival for breast cancer patients from GEO datasets (*P*<0.05, log-rank test).

**Figure 2:**
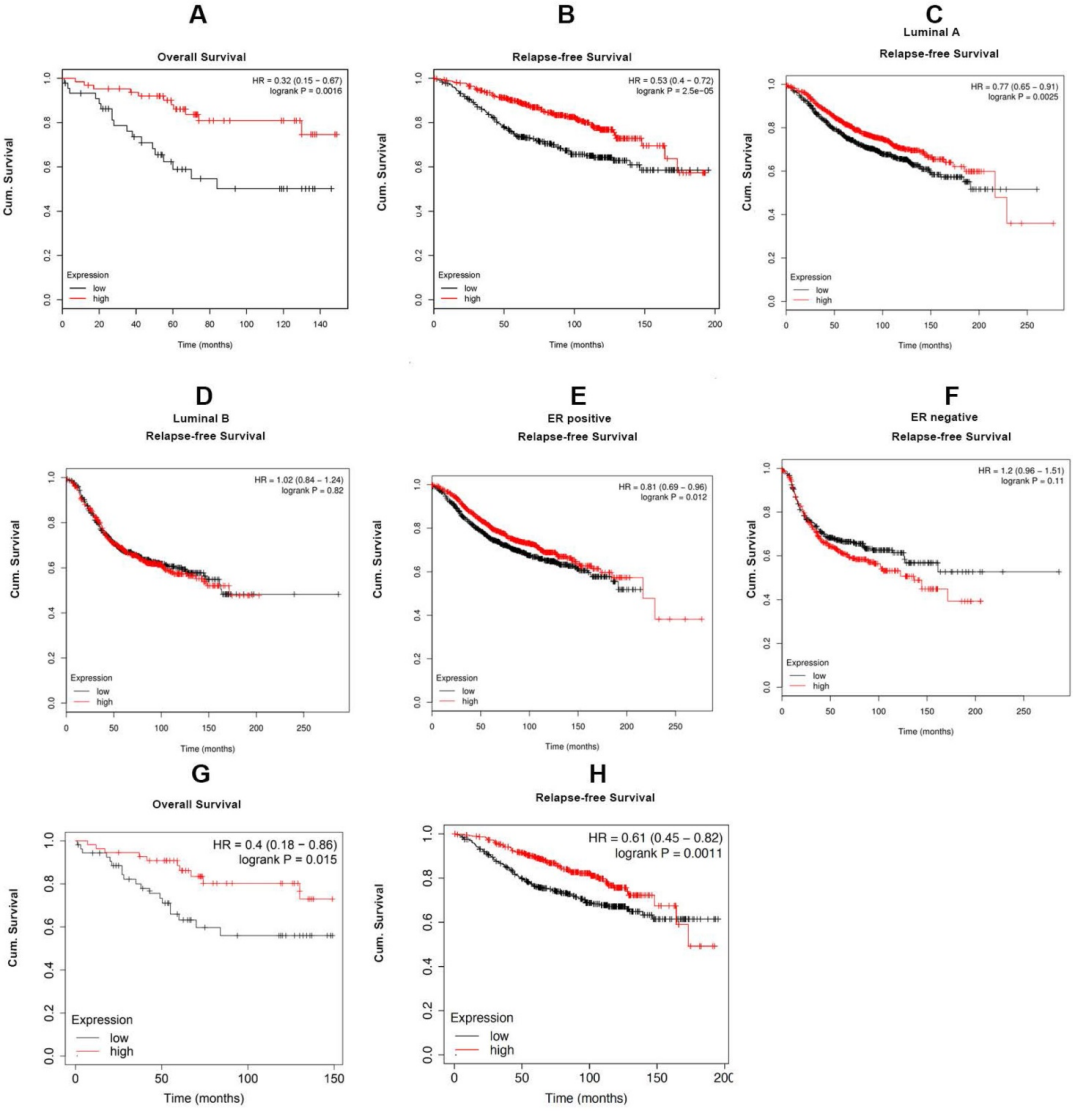
The relationship between Cx43 expression and survival analysis in the subtypes of breast cancer. The correlation of Cx43 expression with survival analysis was analyzed by Kaplan-Meier estimates based on KM plotter online database (www.kmplot.com).

For investigating the role of Cx43 expression in breast cancer, we interrogated the Cancer Genome Atlas (TCGA) database and found that the expression of Cx43 was significantly decreased in primary breast cancer and its subtypes (1097 samples) than that in normal breast samples (114 samples) (Fig. 1A). We then examined the correlation of Cx43 expression with clinicopathological parameters using immunohistochemistry (IHC) in 250 breast cancer samples (tissue microarray). The results showed that low Cx43 expression was correlated with high TNM stage (Fig. 1C). Moreover, low Cx43 expression was markedly correlated with ER and PR negative, but not significantly correlated with HER-2 expression (Fig. 1D-F). Interestingly, low Cx43 level was significantly correlated with high lymph node metastasis (Fig. 1G). Furthermore, the results of Kaplan-Meier survival analysis indicated that low Cx43 expression was correlated with low 10-year overall survival and relapse-free survival (Fig. 1H and I). The relationship between Cx43 expression and survival analysis was further analyzed by Kaplan-Meier estimates based on KM plotter online database (www.kmplot.com) containing 3951 breast cancer samples. The results revealed that low Cx43 expression was correlated with a poor outcome in overall survival and relapse-free survival (Fig. 2A and B). Furthermore, patients with lower expression of Cx43 had greater decreased relapse-free survival in luminal A subtype of breast cancer (Fig. 2C). In addition, in ER^+^ but not ER^-^ patients, there was a significantly lower probability of survival for patients with lower Cx43 expression (Fig. 2E and F).

We next performed a meta-analysis based on TCGA (809 samples) datasets to investigate whether Cx43 expression prognosticates TAM resistance. The results showed that low Cx43 expression in breast cancer was significantly correlated with poor outcome in overall survival and relapse-free survival with restriction of TAM treatment (Fig. 2G and H). We further analyzed the GEO datasets (GSE6532 and GSE2990) of which the patients administrated with TAM monotherapy using Kaplan-Meier survival analysis after each dataset was defined into groups with high and low Cx43 expression 27. The results revealed that the cohorts expressing low level of Cx43 displayed a higher possibility of developing recurrence as compared to the cohorts expressing high level of Cx43 (Fig. 1J and K). The data imply that Cx43 deficiency predicts TAM resistance.

Collectively, these results from both our clinical specimens and database indicate that Cx43 expression serves as a predictor of clinical outcome to TAM therapy.

### Acquired TAM-resistant breast cancer cells exhibited EMT characteristics and Cx43 deficiency

**Figure 3:**
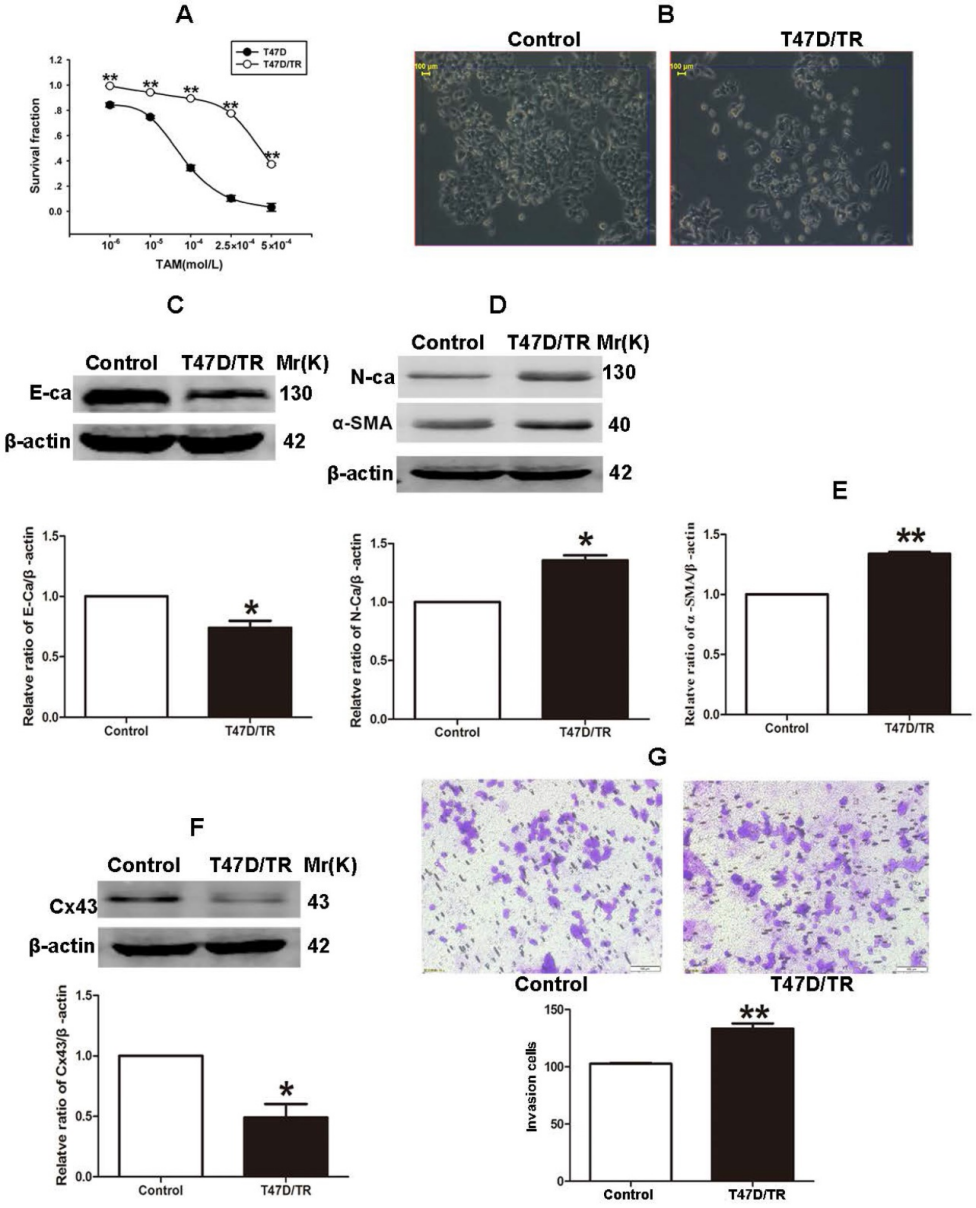
Acquired TAM-resistant breast cancer cells exhibited EMT phenotypes and decreased Cx43 expression. (A) Survival fractions of T47D/TR and parental cells treated with TAM were detected by CCK-8 assay. Data are mean ±SD from 4 independent experiments. **P<0.01 *versus* parental cells. (B) Morphological changes of T47D/TR cells and parental cells. Original magnification, ×400. (C-F) The expressions of E-ca, N-ca, α-SMA, and Cx43 in T47D/TR cells were detected by western blotting assay, respectively. Data are mean ±SD from 3 independent experiments. *P<0.05, **P<0.01 *versus* control group. (G) The invasive ability of T47D/TR cells was detected by transwell assay. Data are mean ±SD from 3 independent experiments. **P<0.01 *versus* control group. Original magnification, ×100.

It has been established that ER^+^ MCF-7 and BT-474 cell lines expressed low level of Cx43, whereas relatively high level of Cx43 was detected in ER^+^ T47D cells 28, 29. Therefore, in order to investigate the role of Cx43 in TAM resistance, T47D cell line was selected to establish acquired TAM-resistant cells (T47D/TR) by continuous exposure to stepwise increasing concentrations of TAM and maintained in 10^-6^ M TAM for at least 18 months. As shown in Fig. 3A, T47D/TR cells exhibited less sensitivity to TAM than their parental cells with IC50 of 4.249×10^-4^ M *versus* 7.02×10^-5^ M, respectively. Moreover, T47D/TR cells showed an elongated, scattered and mesenchymal-like morphology, while their parental T47D cells exhibited tight cell-cell junctions and typical epithelial cobblestone appearance (Fig. 3B). Besides, the expression of E-ca, an epithelial marker, was significantly decreased, whereas the level of mesenchymal markers N-ca and α-SMA were substantially elevated (Fig. 3C-E). The increased invasive capability is considered as an important feature of malignant cells undergoing EMT. As illustrated in Fig. 3G, the invasive potential of T47D/TR cells was greatly enhanced compared with their parental cells. These results suggest a potential role of EMT in acquired TAM resistance of breast cancer cells. Additionally, the expression level of Cx43 was significantly reduced in T47D/TR cells (Fig. 3F), indicating that Cx43 may play a role in reversing EMT and acquired TAM resistance in breast cancer.

### EMT switch modulates Cx43 expression and TAM sensitivity

**Figure 4:**
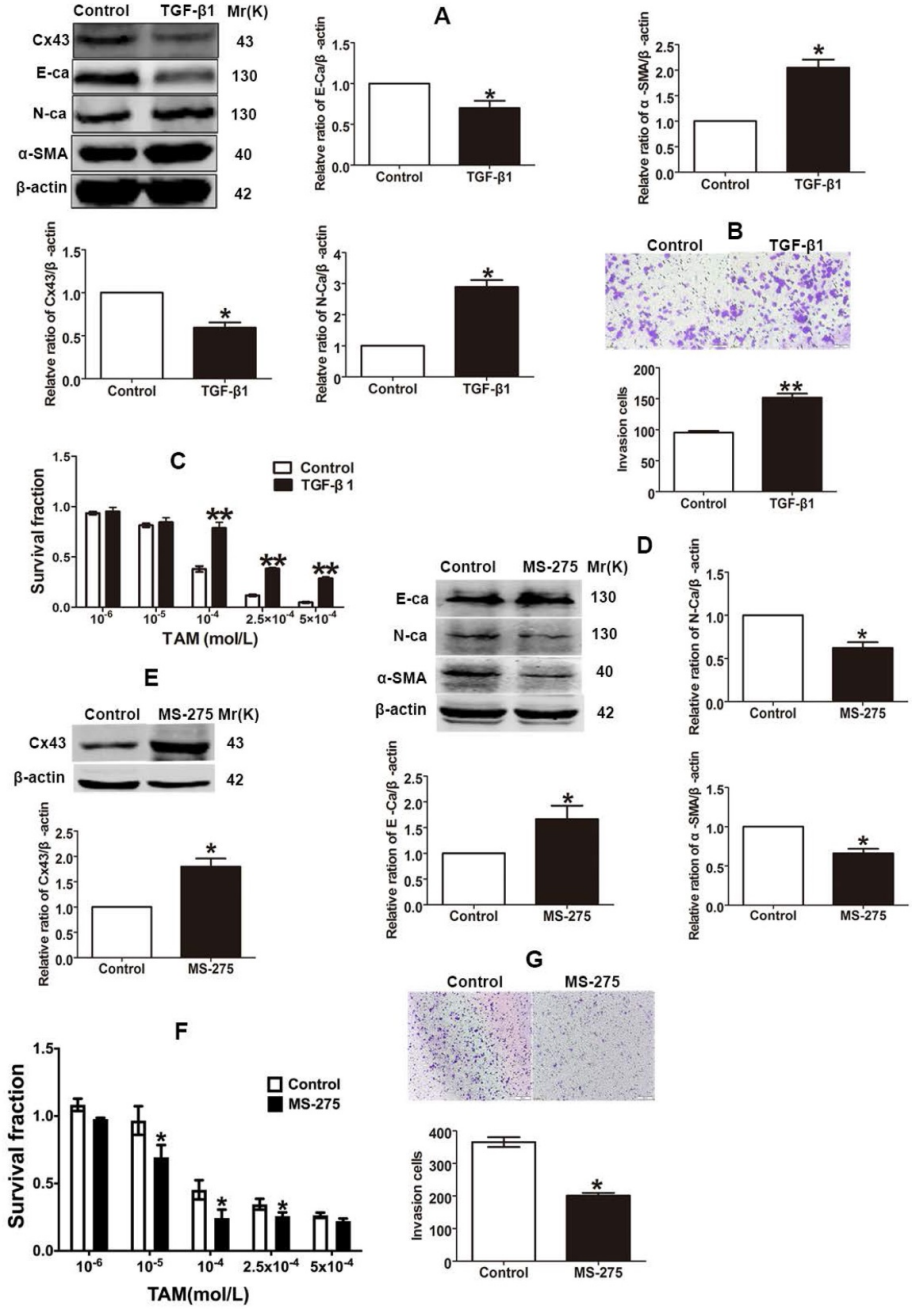
Effects of EMT switch on Cx43 expression and TAM sensitivity in T47D/TS and T47D/TR cells. (A) Effect of TGF-β1 on E-ca, N-ca, α-SMA and Cx43 expression in T47D/TS cells. (B) Effect of TGF-β1 on invasive ability in T47D/TS cells. Original magnification, ×100. (C) Effect of TGF-β1 on TAM sensitivity in T47D/TS cells. (D, E) Effect of MS-275 on E-ca, N-ca, α-SMA and Cx43 expression in T47D/TR cells, respectively. (F) Effect of MS-275 on TAM sensitivity in T47D/TR cells. (G) Effect of MS-275 on invasive ability in T47D/TR cells. Original magnification, ×100. Data are mean ±SD from 3 independent experiments. *P<0.05, **P<0.01 *versus* control group.

The finding that TAM resistant cells exhibited the EMT phenotype suggests that EMT switch may affect TAM sensitivity. Studies showed that TGF-β1 could induce EMT progression via Smad signaling pathway in different types of malignant cells 4, 10. To explore the role of EMT in TAM susceptibility, after T47D/TS cells were treated with TGF-β1, the expression of EMT markers were detected and the cytotoxicity of TAM was measured. As shown in Fig. 4A and B, TGF-β1 repressed E-ca expression, induced N-ca and α-SMA expression and promoted the invasion of T47D/TS cells, indicating that EMT programme was initiated. Moreover, TGF-β1 treatment resulted in a significant downregulation of Cx43 expression and a substantial decrease in TAM cytotoxicity (Fig. 4A and C). The results suggest that EMT induction by TGF-β1 prohibits Cx43 expression and leads to a decrease in TAM sensitivity.

It has been reported that Entinostat (MS-275), a histone deacetylase (HDAC) inhibitor, could trigger the conversion of EMT into mesenchymal-epithelial transition (MET), and thus inhibiting EMT process 30, 31. To explore the role of EMT repression in TAM resistance, after T47D/TR cells were treated with MS-275, the expression of EMT markers and TAM cytotoxicity were measured. As shown in Fig 4D and G, MS-275 induced E-ca expression, suppressed N-ca and α-SMA expression and inhibited the invasion of T47D/TR cells, indicating that MET, a reverse process of EMT, was activated. Additionally, MS-275 treated cells led to the upregulation of Cx43 and exhibited substantially increased sensitivity of T47D/TR cells to TAM (Fig. 4E and F). These results indicate that inhibition of EMT in T47D/TR cells promotes Cx43 expression and reverses TAM resistance.

Collectively, the above results demonstrate that EMT switch modulates Cx43 expression and TAM susceptibility in breast cancer.

### Cx43 reverses TAM sensitivity by attenuating EMT in TAM-sensitive cells in a GJIC-dependent manner

**Figure 5:**
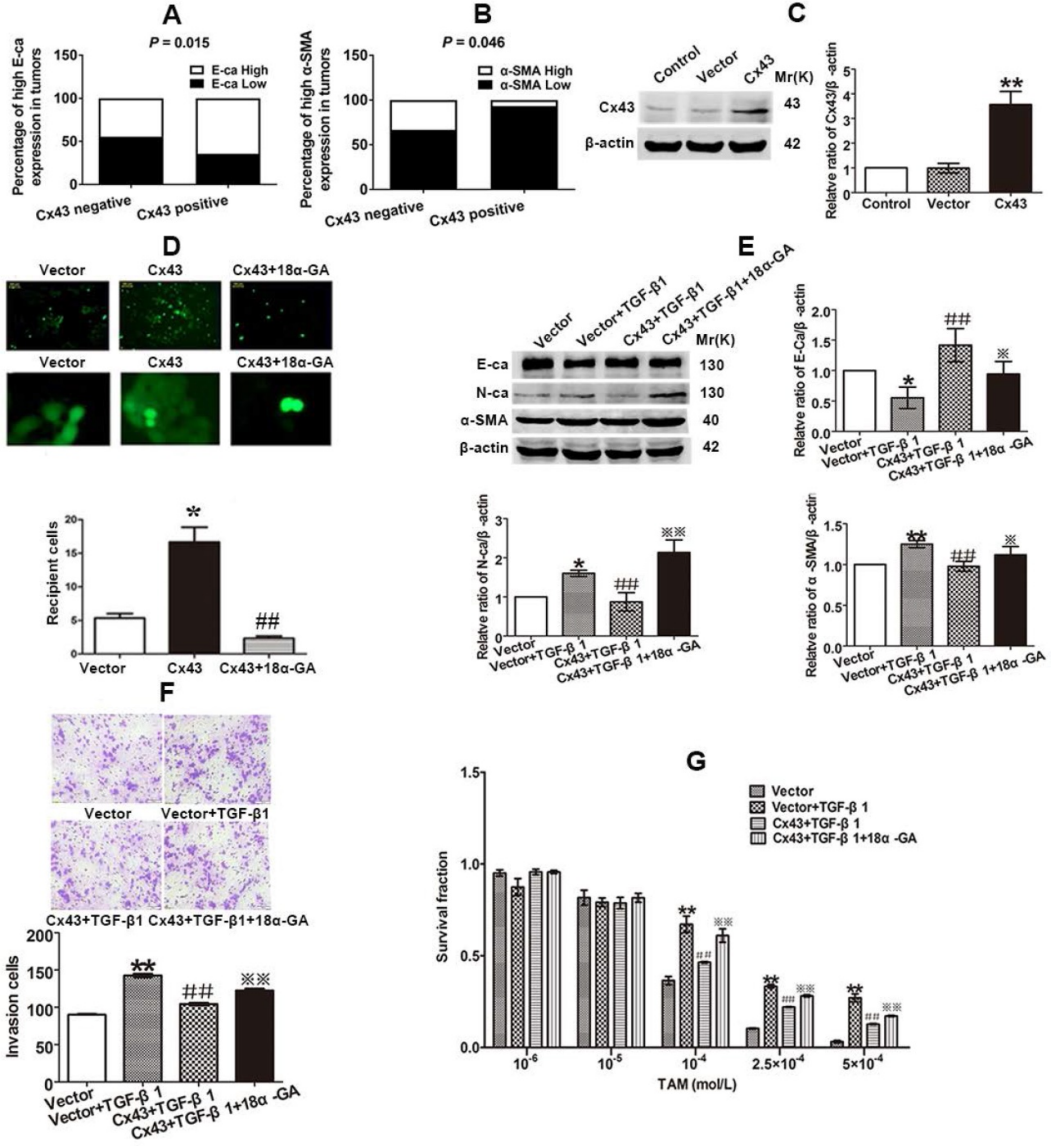
Cx43 suppresses EMT and increases TAM sensitivity in T47D/TS cells in a GJIC-dependent manner. (A, B) Correlation of Cx43 expression with E-ca and α-SMA expression (χ^2^ test). (C) Cx43 overexpression efficacy. (D) Effect of 18α-GA on GJIC determined by “Parachute” dye-coupling assay. (E) Effect of Cx43 overexpression on E-ca, N-ca and α-SMA expression, respectively. (F) Effect of Cx43 overexpression on invasive ability. Original magnification, ×100. (G) Effect of Cx43 overexpression on TAM sensitivity. Data are mean ±SD from 3 independent experiments. *P<0.05, **P<0.01 *versus* vector group, ^##^*P*<0.01, *versus* vector+TGF-β1 group, *P<0.05, **P<0.01, *versus* Cx43+TGF-β1 group. Data are mean ±SD from 3 independent experiments. *P<0.05 *versus* vector group, ^##^*P*<0.01, *versus* Cx43 group.

The findings that EMT modulates Cx43 expression and TAM sensitivity prompt us to bring about a scenario that Cx43 might play a role in modulating EMT and TAM sensibility. We firstly examined the correlation of Cx43 expression and EMT marker E-ca and α-SMA expressions in clinical specimens using IHC. The results showed that high Cx43 expression was positively correlated with E-ca expression, but negatively correlated with α-SMA expression (Fig. 5A and B), indicating that Cx43 may be implicated in regulating EMT process.

We then investigated the role of Cx43 in EMT in breast cancer cells. We firstly built Cx43-overexpressed T47D/TS cells to perform gain-of-function assay (Fig. 5C). As shown in Fig 5E and F, Cx43 overexpression reversed TGF-β1-induced E-ca downregulation, N-ca and α-SMA upregulation and increment of invasive ability, suggesting that overexpression of Cx43 was sufficient to inhibit TGF-β1-induced EMT activation. To determine the role of Cx43 in TGF-β1-incuded TAM resistance, TAM cytotoxicity was measured. As illustrated in Fig 5G, Cx43 overexpression retarded TGF-β1-induced decline in TAM cytotoxicity, indicating that Cx43 overexpression represses TGF-β1-incuded TAM insensitivity.

Since GJIC has been reported to regulate cellular differentiation and chemosensitivity 12, 32, we then investigated whether Cx43-composed GJIC was involved in the TGF-β1-induced EMT and TAM insensitivity. First, parachute assay was performed to assess GJIC after Cx43-overexpressed cells were treated with 10 μM 18a-glycyrrhetinic acid (18α-GA), a non-selective GJIC inhibitor 22. As shown in Fig. 5D, 18α-GA treatment led to reduced GJIC in Cx43-overexpressed T47D/TS cells. Furthermore, we assessed the effect of 18α-GA on EMT and TAM sensitivity in TGF-β1-treated Cx43-overexpressed cells. As illustrated in Fig. 5E and F, 18α-GA treatment reversed TGF-β1-induced reduction of E-ca expression, enhancement of N-ca and α-SMA expression and invasive potential of Cx43-overexpressed cells. Moreover, 18α-GA retarded the inhibitory role of Cx43 in TGF-β1-induced TAM insensitivity (Fig. 5G). These results indicate that GJIC may participate in the reversing effect of Cx43 on TGF-β1-incuded EMT and TAM insensitivity.

**Figure 6:**
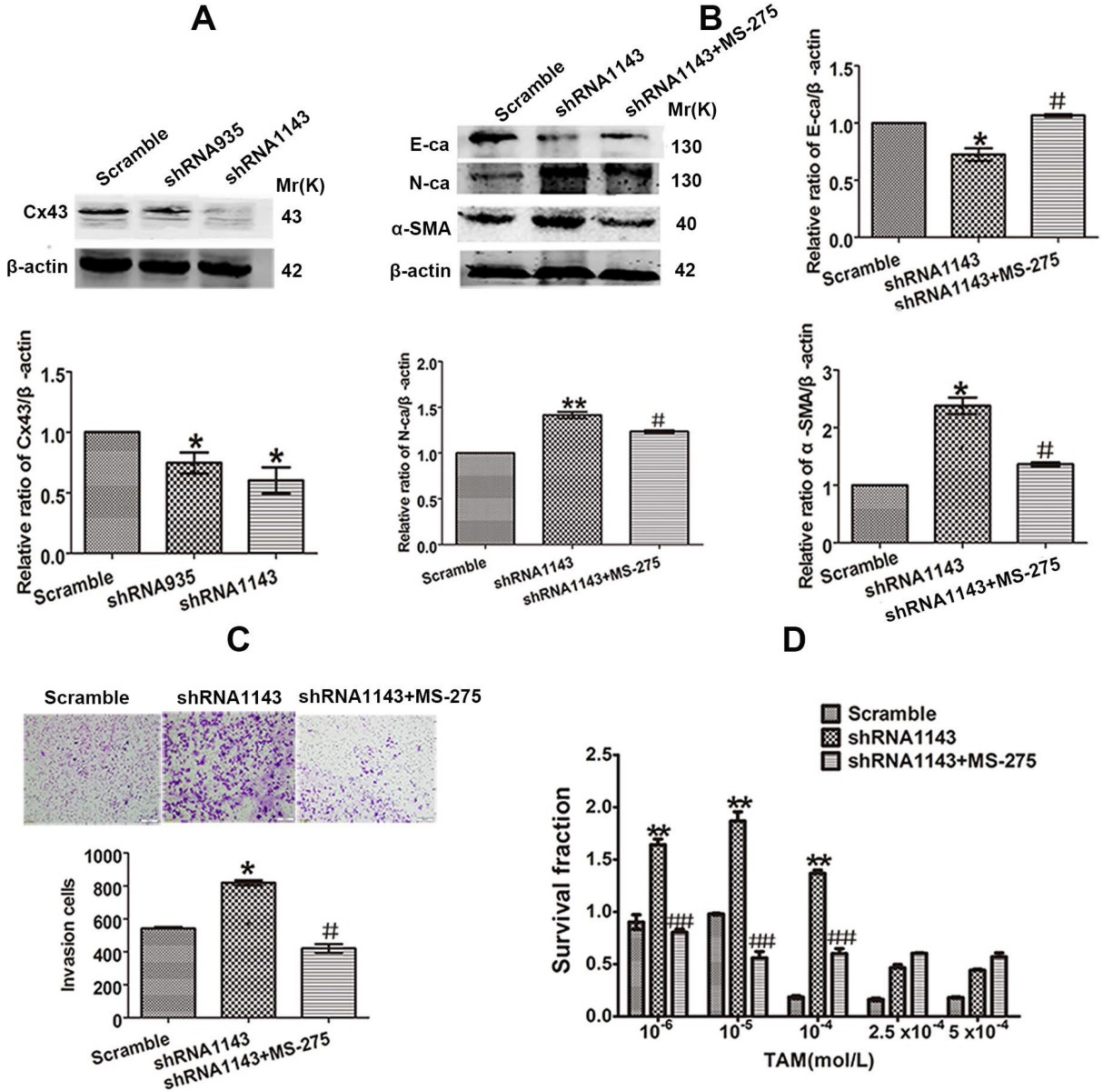
Effect of Cx43 depletion on EMT and TAM sensitivity. (A, B) Effect of Cx43 knockdown on E-ca, N-ca and α-SMA expression. (C) Effect of Cx43 knockdown on invasive ability. Original magnification, ×100. (D) Effect of Cx43 knockdown on TAM sensitivity. Data are mean ±SD from 3 independent experiments. *P<0.05, **P<0.01 *versus* scramble group, ^#^*P*<0.05,^*##*^*P*<0.01 *versus* shRNA1143 group.

For further confirming the role of Cx43 in EMT and TAM sensitivity, loss-of-function assay was performed. We transducted two Cx43 short hairpin RNAs (shRNA, shCx43) into T47D/TS cells and found that shRNA1143 had higher efficacy in Cx43 depletion (Fig. 6A). Thus, shRNA1143 was chosen to build Cx43-depleted T47D/TS cell line. As shown in Fig. 6B-D, Cx43-depleted cells exerted significantly enhanced expression of N-ca and α-SMA, reduced expression of E-ca, increased invasive ability and decreased TAM sensitivity as compared to the cells transfected with scramble shRNA (control group), suggesting a role of Cx43 depletion in EMT activation and TAM insensitivity. Furthermore, MS-275 strongly attenuated Cx43-depletion induced EMT and TAM insensitivity.

Taken together, these results indicate that Cx43 attenuates EMT activation and increases TAM sensitivity in TAM-sensitive cells in a GJIC-dependent manner.

### Cx43 suppresses EMT and increases TAM sensitivity in TAM-resistant cells in a GJIC-independent manner

**Figure 7:**
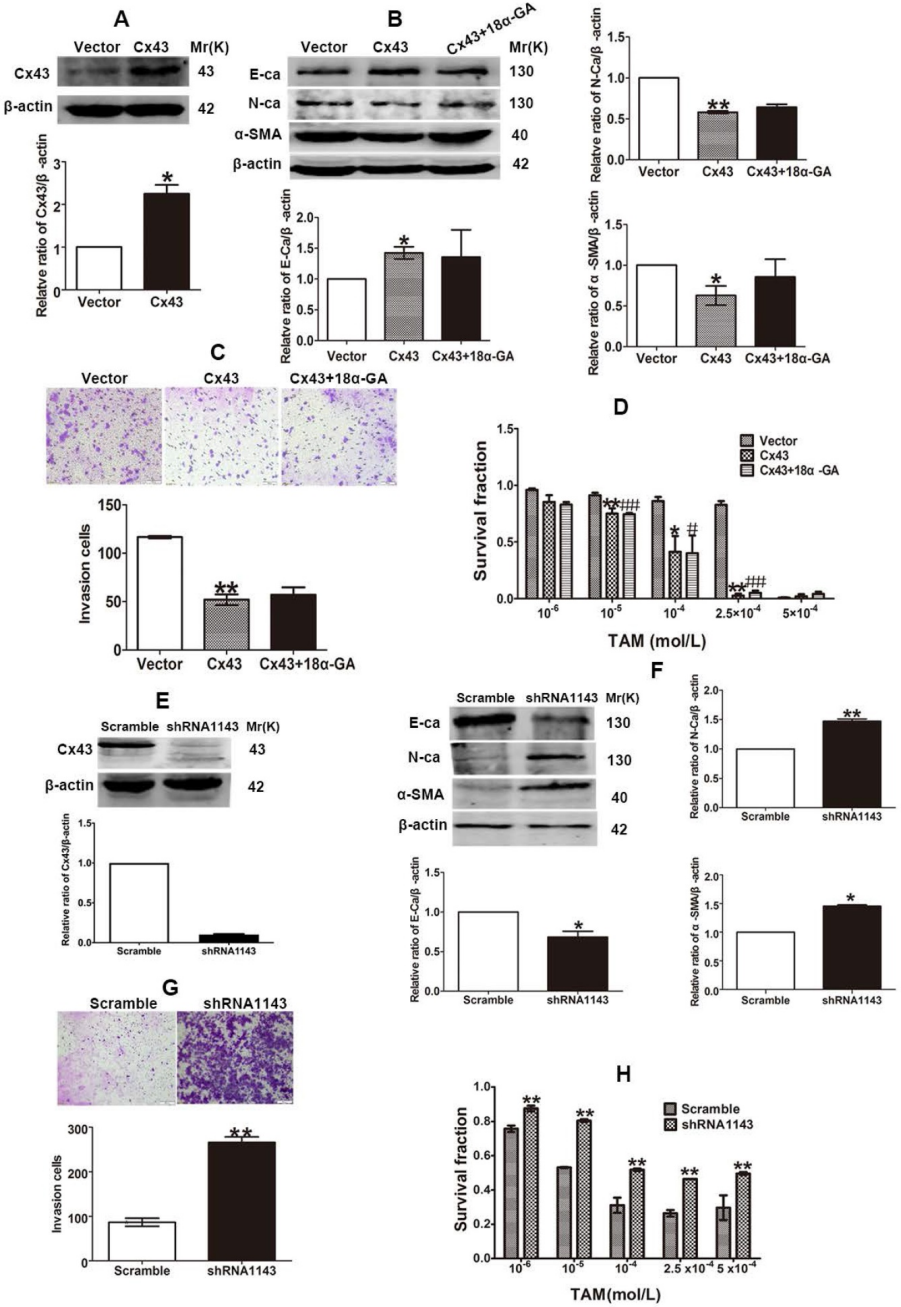
Cx43 reverses TAM resistance by suppressing EMT in acquired T47D/TR cells in a GJIC-independent manner. (A, B) Effect of Cx43 overexpression on E-ca, N-ca and α-SMA expression. (C) Effect of Cx43 overexpression on invasive ability. Original magnification, ×100. (D) Effect of Cx43 overexpression on TAM sensitivity. Data are mean ±SD from 3 independent experiments. *P<0.05, **P<0.01 *versus* vector group, ^#^*P*<0.05, ^##^*P*<0.01, *versus* Cx43 group. (E, F) Effect of Cx43 knockdown on E-ca, N-ca and α-SMA expression. (G) Effect of Cx43 knockdown on invasive ability. Original magnification, ×100. (H) Effect of Cx43 knockdown on TAM sensitivity. Data are mean ±SD from 3 independent experiments. *P<0.05, **P<0.01 *versus* scramble group.

The inhibitory role of Cx43 in EMT and TAM sensitivity in T47D/TS cells indicates that Cx43 may play a role in reversing TAM resistance. In order to investigate the effect of Cx43 in EMT and TAM resistance, we established Cx43-overexpressed and -silenced T47D/TR cell lines, and EMT and TAM sensitivity were evaluated in these two cell lines. The results showed that overexpression of Cx43 significantly enhanced E-ca expression, inhibited N-ca and α-SMA expression and invasive ability of T47D/TR cells (Fig. 7A-C). Whereas knockdown of Cx43 expression remarkably attenuated E-ca expression, increased N-ca and α-SMA expression and elevated invasive potential of T47D/TR cells (Fig. 7E-G), suggesting that Cx43 suppresses EMT in acquired TAM-resistant cells. Besides, Cx43 overexpression improved TAM sensitivity, while Cx43 depletion repressed TAM sensitivity in T47D/TR cells (Fig. 7D and H). These results indicate that Cx43 is sufficient to reverse TAM resistance.

To explore the effect of GJIC on Cx43-meidated reversion of EMT and TAM resistance, we assessed the role of 18α-GA in EMT and TAM cytotoxicity in Cx43-overexpressed T47D/TR cells. The results showed that pretreating cells with 10 μM 18α-GA, a GJIC inhibitor shown to remarkably suppress GJIC at this concentration (Fig. 5D), did not cause a significant difference in EMT and TAM sensitivity (Fig. 7B-D). The results suggest that GJIC was not involved in Cx43-mediated reversion of EMT and TAM resistance in T47D/TR cells.

Collectively, the above results suggest that Cx43 reverses TAM resistance by attenuating EMT in acquired TAM-resistant cells in a GJIC-independent manner.

### Cx43 depletion induces TAM insensitivity in T47D/TS cells transplanted xenograft tumor model

**Figure 8:**
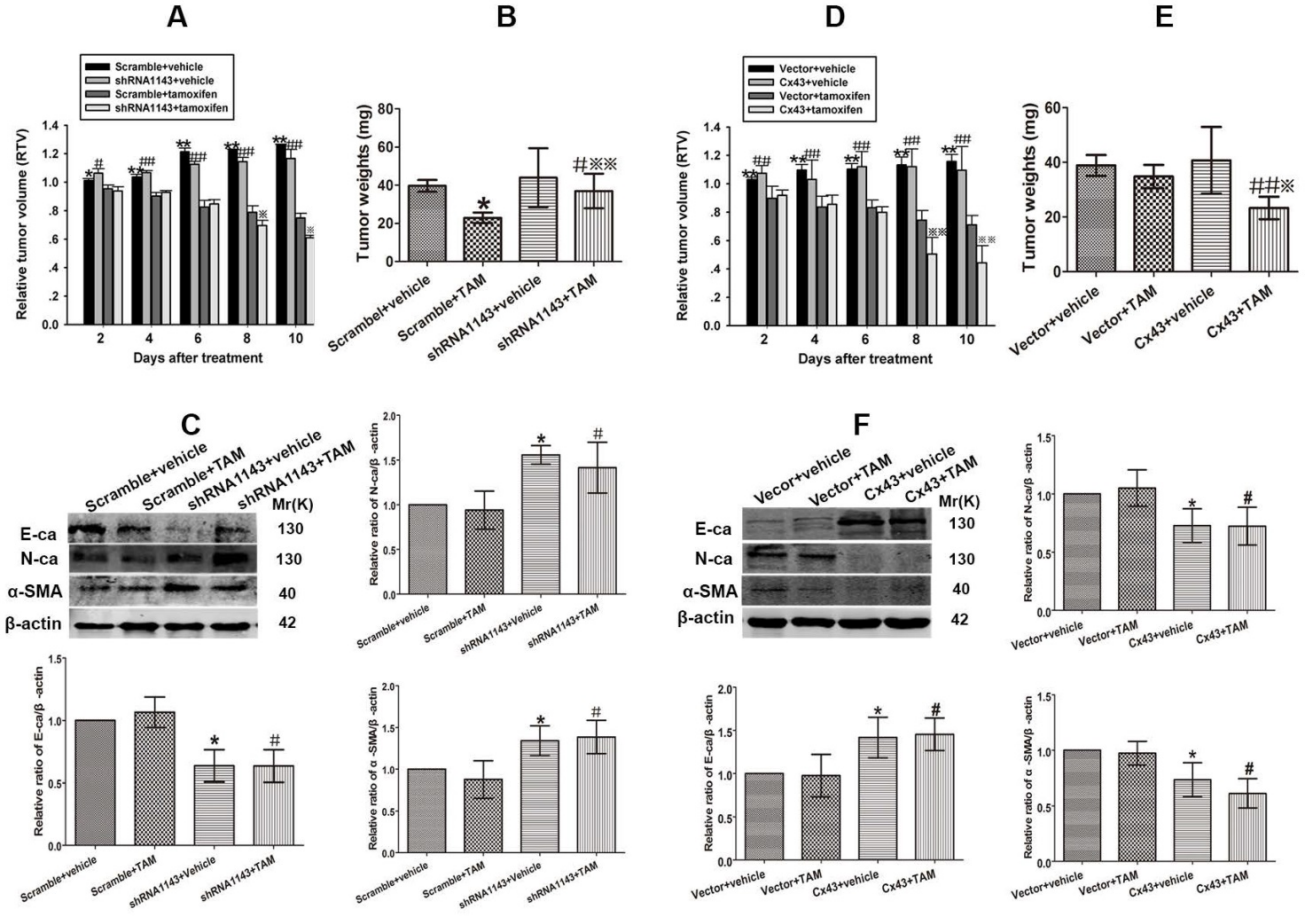
Effect of Cx43 expression on TAM sensitivity in xenograft tumors derived from T47D/TR and T47D/TS cells. (A) Effect of Cx43 depletion on the relative tumor volume (RTV) of xenograft transplanted by T47D/TS cells after TAM treatment. Data are mean ±SD from 4-8 independent experiments. *P<0.05, **P<0.01 *versus* scramble+TAM group, ^#^*P*<0.05, ^##^*P*<0.01 *versus* shRNA1143+TAM group, *P<0.05 *versus* scramble+TAM group. (B) Effect of Cx43 depletion on tumor weights of xenograft transplanted by T47D/TS cells after TAM treatment. (C) Expression of E-ca, N-ca and α-SMA of T47D/TS cells transplanted xenograft after TAM treatment. Data are mean ±SD from 4-8 independent experiments. *P<0.05 *versus* scramble+vehicle group, ^#^*P*<0.05 *versus* shRNA1143+vehicle group, **P<0.01 *versus* scramble+TAM group. (D) Effect of Cx43 overexpression on the RTV of xenograft transplanted by T47D/TR cells after TAM treatment. Data are mean±SD from 4-8 independent experiments. **P<0.01 *versus* vector+TAM group, ^##^*P*<0.01 *versus* Cx43+TAM group, **P<0.01 *versus* vector+TAM group. (E) Effect of Cx43 overexpression on tumor weights of xenograft transplanted by T47D/TR cells after TAM treatment. ^##^*P*<0.01 *versus* vector+TAM group, *P<0.05 *versus* Cx43+vehicle group. (F) Expression of E-ca, N-ca and α-SMA of xenograft-transplanted T47D/TR cells after TAM treatment. Data are mean±SD from 4-8 independent experiments. *P<0.05 *versus* vector+vehicle group, ^#^*P*<0.05, ^##^*P*<0.01 *versus* Cx43+vehicle group, *P<0.05 *versus* vector+TAM group.

To investigate the role of Cx43 in modulating TAM sensitivity *in vivo*, Cx43-deficient and its scramble T47D/TS cells were transplanted into BALB/c-nude mice. The xenograft-bearing mice were administrated with TAM citrate (30 mg/kg per day, gavaged orally) for 10 days, and the mean RTV was calculated for each treatment group. As shown in Fig. 8A, TAM treatment suppressed tumor growth in both Cx43-deficient and scramble xenografts. Importantly, the inhibitory effects of TAM on the scramble xenografts were more dramatic due to a substantial decrease in their mean RTV and tumor weights, than Cx43-deficient xenografts (Fig. 8A and B). Meanwhile, the expression of EMT biomarkers were detected after treatment. The results showed that E-ca expression was significantly attenuated, while N-ca and α-SMA expressions were greatly increased in Cx43-deficient xenografts compared with scramble xenografts (Fig. 8C). The results indicate that Cx43 depletion facilitates TAM insensitivity via inducing EMT *in vivo*.

### Cx43 overexpression reverses TAM resistance in T47D/TR cells transplanted xenograft tumor model

We next explored the role of Cx43 in reversing TAM resistance* in vivo*. Cx43-overexpressed and its control T47D/TR cells were used to establish xenograft bearing mouse model. The mice were gavaged orally with 30 mg/kg TAM citrate for 10 days. As illustrated in Fig. 8D, TAM treatment prohibited tumor growth in both Cx43-overexpressed and control xenografts. Notably, Cx43-overexpressed xenografts exhibited more sensitivity to TAM as compared to control xenografts due to greatly reduced mean RTV and tumor weights in Cx43-overexpressed xenografts than control xenografts (Fig. 8D and E). Additionally, Cx43 overexpression could significantly augment E-ca expression and inhibit N-ca and α-SMA expression in xenograft tumors (Fig. 8F). These results suggest that Cx43 overexpression reverses TAM resistance via inhibiting EMT *in vivo*.

### Reciprocal regulation between Cx43 and PI3K/Akt pathway is involved in Cx43-mediated repression in EMT and TAM resistance

**Figure 9:**
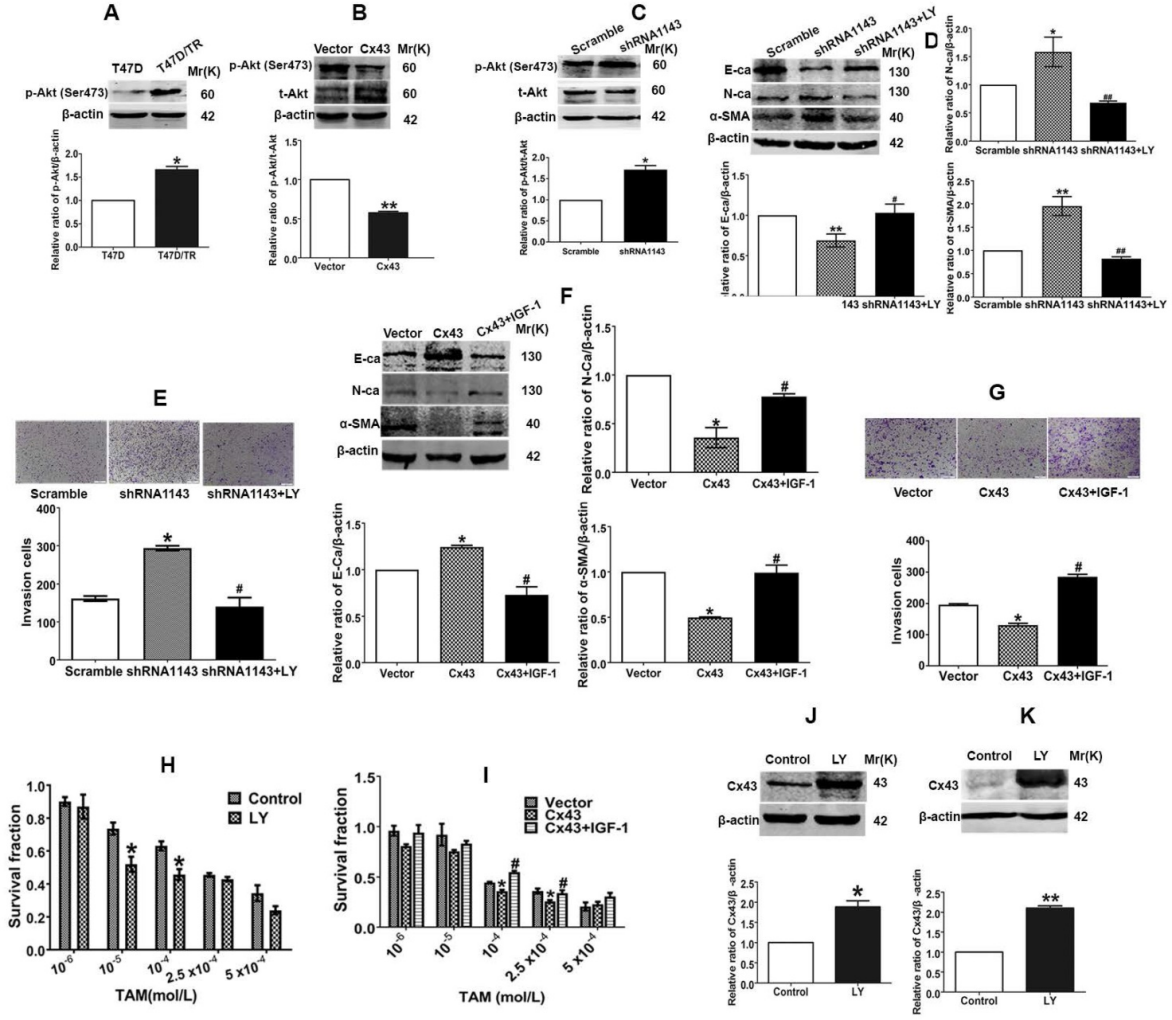
Reciprocal regulation between Cx43 and PI3K/Akt pathway is involved in Cx43-mediated repression in EMT and TAM resistance. (A) Akt activity of T47D/TR and parental cells. (B, C) Effects of Cx43 overexpression and knockdown on Akt activity in T47D/TR cells. (D) Effect of LY294002 on E-ca, N-ca and α-SMA expression in Cx43-depleted T47D/TR cells. (E) Effect of LY294002 on invasive ability in Cx43-depleted T47D/TR cells. Original magnification, ×100. (F) Effect of LY294002 on TAM sensitivity in Cx43-depleted T47D/TR cells. (G) Effect of IGF-1 on E-ca, N-ca and α-SMA expression in Cx43-overexpressed T47D/TR cells. (H) Effect of IGF-1 on invasive ability in Cx43- overexpressed T47D/TR cells. Original magnification, ×100. (I) Effect of IGF-1 on TAM sensitivity in Cx43- overexpressed T47D/TR cells. (J, K) Effect of Akt activity on Cx43 expression in T47D/TR and T47D/TS cells, respectively. Data are mean±SD from 3 independent experiments. *P<0.05, **P<0.01 *versus* control, vector or scramble group, ^#^*P*<0.05, ^##^*P*<0.01 *versus* shRNA1143 group.

According to the above results, we became interested in investigating the underlying molecular mechanism of Cx43-mediated repression of EMT and reversion of TAM resistance in breast cancer cells. It has been proven that PI3K/Akt pathway plays a vital role in activating EMT and drug resistance in malignancies 33, 34. There are reports showing that Cx43 could activate PI3K/Akt signaling and PI3K/Akt signaling could increase Cx43 phosphorylation in cardiomyocytes 35, 36. Thus, we hypothesized that there may be a mutual regulation between Cx43 and PI3K/Akt pathway in EMT and acquired TAM resistance. As illustrated in Fig. 9A, p-Akt expression was remarkably increased in acquired T47D/TR cells compared with their parental cells. Overexpressing Cx43 could significantly reduce Akt phosphorylation, while depleting Cx43 expression could greatly elevate Akt phosphorylation in T47D/TR cells (Fig. 9B and C). These results indicate that Cx43 is sufficient to retard PI3K/Akt signaling activation.

We next explored the role of PI3K/Akt signaling in Cx43-mediated EMT repression. As illustrated in Fig. 9D and E, treatment of Cx43-depleted T47D/TR cells with a specific PI3K/Akt signaling inhibitor LY294002 could attenuate the facilitating effects of Cx43 depletion on EMT, as represented by the elevated E-ca expression, decreased N-ca and α-SMA expression and attenuated invasive capability as compared to LY294002-untreated cells. Whereas, pretreatment of Cx43-overexpressed T47D/TR cells with PI3K/Akt signaling activator IGF-1 remarkably eliminated the inhibitory effects of Cx43 overexpression on EMT (Fig. 9F and G). Furthermore, LY294002-treated Cx43-depleted T47D/TR cells exhibited more sensitivity to TAM compared with LY294002-untreated cells (Fig. 9H), while IGF-1-treated Cx43-overexpressed T47D/TR cells showed less TAM sensitivity compared to IGF-1-untreated cells (Fig. 9I). The results imply that PI3K/Akt signaling is required for Cx43-mediated reversion of EMT and TAM resistance.

We then investigated whether PI3K/Akt pathway was involved in Cx43 expression. As shown in Fig. 9J and K, treatment with LY294002 resulted in substantial increased Cx43 expression in both T47D/TR and T47D/TS cells, indicating that PI3K/Akt signaling is sufficient to attenuate Cx43 expression.

Taken together, the above findings suggest that the functional interplay between Cx43 and PI3K/Akt pathway contributes to the acquired TAM resistance in breast cancer cells via the induction of EMT.

### Cx43 and c-Src interaction leads to inactivation of PI3K/Akt signaling and attenuation of EMT and TAM resistance

**Figure 10:**
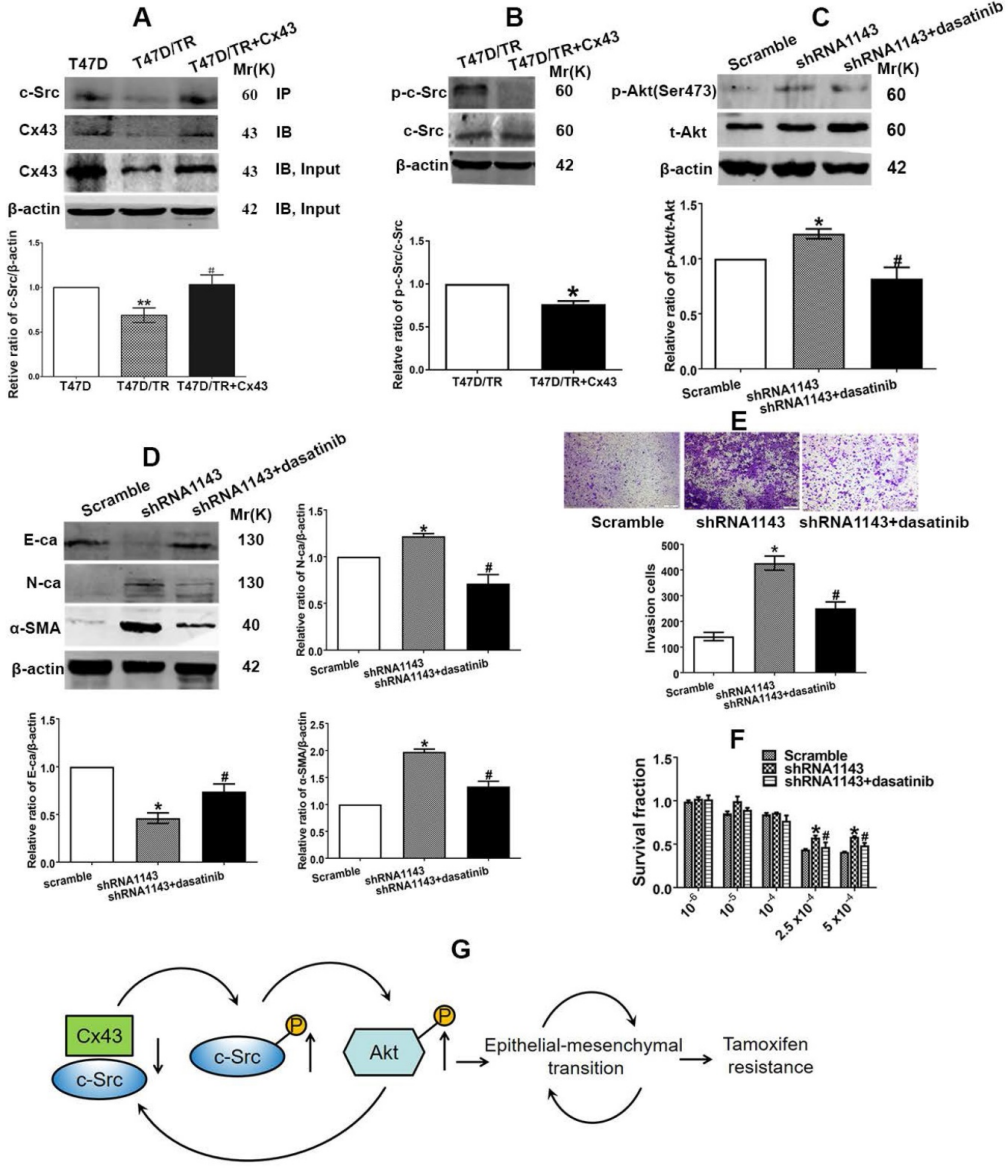
Cx43 and c-Src interaction leads to inactivation of PI3K/Akt signaling and attenuation of EMT and TAM resistance**.** (A) The interaction of Cx43 with c-Src was detected using Co-IP assay. (B) c-Src activity of T47D/TR cells. (C) Akt activity of Cx43-depleted T47D/TR cells after cells were treated with dasatinib. (D) Effect of dasatinib on E-ca, N-ca and α-SMA expression in Cx43-depeleted T47D/TR cells. (E) Effect of dasatinib on invasive ability in Cx43-depeleted T47D/TR cells. Original magnification, ×100. (F) Effect of dasatinib on TAM sensitivity in Cx43-depeleted T47D/TR cells. Data are mean±SD from 3 independent experiments. *P<0.05, **P<0.01 *versus* T47D, T47D/TR or scramble group, ^#^*P*<0.05, ^##^*P*<0.01 *versus* T47D/TR or shRNA1143 group. (G) Model of feedback loop contributing to TAM resistance. The regulatory circuit impedes Cx43 expression, leading to an impairment of the interaction of Cx43 with c-Src and subsequently activation of PI3K/Akt pathway, which further retards Cx43 expression, thereby augmenting EMT and TAM resistance in breast cancer.

We then became interested in investigating the mechanism of which Cx43 retards PI3K/Akt pathway in breast cancer cells. It has been documented that the intracellular carboxy tail of Cx43 interacts with multiple signaling and scaffolding proteins, thereby modulating the intercellular signaling 37. We next sought to predict the downstream target protein that could potentially interact with Cx43 and preferably associate with regulation of PI3K/Akt pathway and TAM resistance using the STRING database (http://string-db.org/) 38 and the Human Protein Reference Database (HPRD) (http://www.hprd.org/) 39. We found that Cx43 could potentially interact with c-Src, a member of the Src family of non-receptor tyrosine kinases, via its SH3-binding domain. c-Src has been reported to play vital roles in EMT, chemotherapy resistance and activate PI3K/Akt pathway 40, 41. Therefore, we expected that the interaction between Cx43 and c-Src might play a role in the activation of PI3K/Akt pathway, subsequently affect EMT and TAM resistance. We firstly determined the interaction of Cx43 with c-Src. The results of Co-IP assay revealed that Cx43 could interact with c-Src (Fig. 10A). Interestingly, impaired Cx43 and c-Src interaction was observed in T47D/TR cells than their parental T47D cells (Fig. 10A). Additionally, exogenously expressing Cx43 in T47D/TR cells suppressed the phosphorylation of c-Src (Fig. 10B), implying that Cx43 and c-Src interaction attenuates c-Src activation.

We next detected whether c-Src affects the activation of PI3K/Akt signaling in T47D/TR cells. As illustrated in Fig. 10C, treatment of Cx43-depleted T47D/TR cells with c-Src inhibitor dasatinib could attenuate the facilitating effect of Cx43 depletion on Akt phosphorylation, indicating that c-Src participates in Cx43-mediated inhibition of Akt activation.

We then examined whether c-Src participated in Cx43-meidated repression of EMT and TAM resistance. As shown in Fig 10D and E, treatment of Cx43-depleted T47D/TR cells with c-Src inhibitor dasatinib could attenuate the facilitating role of Cx43 depletion in EMT as represented by elevated E-ca expression and decreased N-ca, α-SMA expression and invasive ability. Additionally, dasatinib-treated Cx43-depleted T47D/TR cells showed more sensitivity to TAM compared with dasatinib-untreated cells (Fig. 10F). These data indicate that c-Src is required for Cx43-mediated inhibition in EMT and TAM resistance.

In summary, the above data indicate that Cx43 and c-Src interaction suppresses c-Src activation, leading to the inactivation of PI3K/Akt signaling, thereby attenuating EMT and TAM resistance.

## Discussion

Endocrine therapy plays a vital role in improving the prognosis of ER^+^ breast cancer. TAM is an important antiestrogen drug that can improve the 5-year overall survival and disease-free survival of ER^+^ breast cancer patients 42. Nevertheless, *de novo* and acquired TAM resistance remains a major therapeutic challenge 43. Therefore, it is critical to identify novel therapeutic targets or more specific biomarkers predicting the therapeutic response to TAM.

Here, we characterized a critical role of Cx43 in TAM resistance. We found that Cx43 expression was downregulated in acquired TAM-resistant breast cancer cells. Cx43 overexpression or combination treatment with dasatinib improved TAM susceptibility, while Cx43 depletion induced TAM resistance, in T47D/TS and T47D/TR cells and xenograft tumors (Figs. 4-10). These results demonstrate that Cx43 could serve as an attractive target for overcoming TAM resistance and suggest that therapeutic strategies designed to maintain or enhance Cx43 expression may augment the efficacy of TAM treatment.

In the present study, we also found that high Cx43 expression was correlated with low TNM stage, low lymph node metastasis and high 10-year overall survival and relapse-frees survival in the entire patient cohort and the subtypes of ER^+^, basal and luminal A (Fig. 1B-K), indicating that Cx43 serves as an independent prognostic marker for breast cancer patients. These findings are consistent with a recent study that elevating Cx43 protein levels were linked with significantly improved metastasis-free survival 44. Notably, data mining analysis of TCGA and several GEO datasets in the cohort of breast cancer patients who received TAM monotherapy revealed that high Cx43 expression was highly associated with improved outcome in relapse free survival (Fig. 1J and K, Fig. 2), suggesting that Cx43 may serve as a potential prognostic marker for the therapeutic response to TAM treatment.

The present study makes clear that there is a significant Cx43-dependent component of TAM susceptibility, indicating that Cx43 expression in breast cancer is an important determiner of the clinical response to TAM treatment. Agents affecting Cx43 expression may influence the clinical response to TAM-based endocrinotherapy. It has been proven that several agents, including all-trans retinoic acid (ATRA) and resveratrol, increase the expression of Cxs including Cx43, causing an enhancement of antineoplastic efficacy 45, 46. Thus, the efficacy of TAM therapy could be increased in the event of TAM treatment used concurrently with these agents in clinical settings. It should be noted, however, that several platinum-based chemotherapeutics, such as cisplatin and oxaliplatin, have been reported to inhibit Cx43 expression 47. Therefore, if TAM therapy is used concurrently with platinum-based chemotherapy, the antitumor efficiency of TAM might be declined, probably leading to TAM resistance, by the suppression of Cxs expression caused by platinum-based reagents.

EMT is integral in wound healing, cell behavior and pathologically contributes to cancer progression and chemotherapeutic resistance 48. Reports have indicated that TAM-resistant MCF-7 breast cancer cells undergo EMT phenotype 8, 49. In the present study, we observed that T47D/TR cells exerted EMT phenotype (Fig. 3B-E and G). Induction EMT of T47D/TS cells by TGF-β1 could attenuate TAM sensitivity (Fig. 4A and B), whereas repression EMT of T47D/TR cells by MS-275 could reverse TAM resistance (Fig. 4D and G). These results are consistent with previous studies which indicate that EMT is a key event to confer TAM resistance 50, 51. We next investigated whether Cx43 reversed TAM resistance via retarding EMT. We found that high Cx43 expression was significantly correlated with high E-ca expression and low α-SMA expression in clinical specimens (Fig. 5A and B) and Cx43 was sufficient to alleviate TAM resistance by impeding EMT in TAM-sensitive and -resistant breast cancer cells and in xenografts (Fig. 5E-G, Fig 8). These results suggest the possibility that attenuating EMT by up-regulation or maintenance of Cx43 expression may be a novel therapeutic strategy to overcome TAM resistance.

GJIC-dependent effects of Cxs, including Cx32, Cx43 and Cx26, on the sensitivity of antineoplastics have been reported in many studies 22, 52, 53. In the present study, we observed that 18α-GA, a well-defined GJIC inhibitor, restored the inhibitory role of Cx43 in TGF-β1-induced EMT and TAM insensitivity in TAM-sensitive cells (Fig. 5D-G), indicating a GJIC-dependent role of Cx43 in reversing TGF-β1-induced EMT and TAM insensitivity in TAM-sensitive cells. It has been well established that GJIC-mediated effects on the sensitivity of antineoplastic agents depend on the transfer of toxic substances from one cell to an adjacent other via GJ channels 11. It would be interesting to investigate the substances penetrating via GJ channels responsible for TGF-β1-induced TAM sensitivity in further study. In spite of GJIC-dependent role of Cxs in chemosensitivity, GJIC-independent effects of Cxs on the resistance of antineoplastic agents have also been reported recently. For example, Cx26 protein itself, which could not form GJIC, could promote gefitinib resistance in NSCLC cells 54. Additionally, Cx43 prohibited cisplatin resistance in a GJIC-independent manner 55. In the present study, we found that GJIC inhibitor 18α-GA could not suppress the inhibitory role of Cx43 in EMT and TAM sensitivity in TAM-resistant cells (Fig. 7B-D), suggesting that Cx43-mediated reversion of EMT and TAM resistance is independent of GJIC.

PI3K/Akt signaling-dependent EMT has been reported to facilitate resistance to chemotherapy in human multiple myeloma 56, cisplatin resistance in hepatocellular carcinoma cells 57 and TAM resistance in breast cancer cells 58. Reports have demonstrated that Cx43 could activate PI3K/Akt signaling in cardiomyocytes 36 and PI3K/Akt signaling activation facilitates Cx43 expression in myocardial ischemia-reperfusion injury 59. Thus, we hypothesized that there might exist a mutual regulation of PI3K/Akt signaling and Cx43 expression in acquired endocrine treatment resistance. In the study, we found that PI3K/Akt pathway was activated in acquired T47D/TR cells (Fig. 9A). Exogenous Cx43 expression could prevent PI3K/Akt signaling activation, while Cx43 depletion could activate Akt in T47D/TR cells (Fig. 9B and C). Moreover, suppression of PI3K/Akt by specific inhibitor LY294002 could attenuate the facilitating effects of Cx43 depletion on EMT and TAM resistance, whereas pretreatment of Cx43-overexpressed T47D/TR cells with PI3K/Akt signaling activator IGF-1 remarkably eliminated the inhibitory effects of Cx43 overexpression on EMT and TAM resistance in acquired T47D/TR cells (Fig. 9D-I). These data suggest that PI3K/Akt pathway is required for Cx43-mediated inhibition of EMT and TAM resistance. Interestingly, we also found that inhibition of PI3K/Akt pathway led to decreased Cx43 expression in T47D/TR and their parental cells (Fig. 9J and K). However, the underlying mechanism was not investigated in the study. There are reported showing that activation of PI3K/Akt signaling by shear stress resulted in elevated accumulation of β-catenin in nucleus, which could elevate Cx43 expression by binding to the Cx43 promoter 60. Further study should be designed to investigate whether β-catenin was involved in the elevation of Cx43 expression by PI3K/Akt pathway. Collectively, these data indicate that there exists a feedback modulation between Cx43 expression and PI3K/Akt pathway, retarding Cx43 expression and activating PI3K/Akt signaling, subsequently contributing to EMT and TAM resistance in breast cancer.

The carboxy tail of Cx43 has been reported to contain multiple domains interacting with scaffolding proteins 61. We then predicted the proteins that could potentially interact with Cx43 and preferably regulate PI3K/Akt pathway and TAM resistance using STRING and HPRD databases. c-Src was selected as a downstream protein of Cx43 since c-Src has been reported to activate PI3K/Akt pathway by directly phosphorylating its p85 regulatory subunit 40, 62 and participate in EMT and the resistance of chemotherapy and endocrinotherapy 63, 64. We hypothesized that Cx43 and c-Src interaction might modulate c-Src activity, thereby regulating the activation of PI3K/Akt pathway. Co-IP assays directly support the interaction of Cx43 with c-Src (Fig. 10A). Western blotting assay revealed that overexpression of Cx43 sufficiently inhibited c-Src phosphorylation (Fig. 10B), indicating that the interaction of Cx43 with c-Src reduces c-Src activity, which is consistent with the result that Cx43 and c-Src interaction suppressed c-Src activity in glioma stem cells 65. In addition, Co-IP assay revealed that Cx43 and c-Src interaction was significantly impaired in T47D/TR cells than their parental T47D cells. Moreover, inhibition of c-Src activity by dasatinib in Cx43-depelted cells attenuated the facilitating role of Cx43 depletion in EMT and TAM resistance (Fig 10D~F), indicating that Cx43 and c-Src interaction deficit contributes to EMT and TAM resistance. We then investigated whether c-Src was involved in Cx43-mediated modulation of PI3K/Akt signaling. The results showed that suppressing c-Src activity by dasatinib attenuated the enhanced role of Cx43 depletion in Akt phosphorylation (Fig. 10C), implying the participation of c-Src in Cx43-mediated inhibition of Akt activation. Collectively, the above data indicate that Cx43 and c-Src interaction suppresses c-Src activation, leading to the inactivation of PI3K/Akt signaling, thereby attenuating EMT and TAM resistance.

It is worth noting that a novel feedback regulation between the interaction of Cx43 with c-Src and PI3K/Akt pathway conferring TAM resistance was found in the study. The regulatory circuit impedes Cx43 expression, leading to an impairment of the interaction of Cx43 with c-Src and subsequently activation of PI3K/Akt pathway, which further retards Cx43 expression, thereby inducing EMT and TAM resistance in breast cancer (Fig. 10G). Therefore, therapeutic strategies designed to disturb the regulatory loop may be beneficial to interrupt EMT and TAM resistance. There are reports showing that cell-penetrating peptides synthesized according to the sequences of Cx43 containing c-Src binding sites could reduce c-Src activity and reverse EMT in glioma stem cells 65. Accordingly, one would expect cell-penetrating peptide to reverse EMT and TAM resistance by disturbing the feedback loop. It is necessary to investigate the role of cell-penetrating peptide in EMT and TAM sensitivity in our further studies.

In summary, the work reported here demonstrates that Cx43 deficiency confers TAM resistance by inducing EMT via activating c-Src/PI3K/Akt pathway. This brings about several clinical considerations. Firstly, pharmacologic strategies designed to reinforce Cx43 function can sensitize breast cancer cells to endocrine therapy. Oppositely, factors that suppress the functionality of Cx43 may lead to endocrine treatment resistance in breast cancer, causing substantial declined therapeutic efficacy. Secondly, the assessment of Cx43 expression may be used to categorize patients in accordance with their benefits from TAM treatment, which may offer patients a better choice for TAM and a more personalized treatment approach.
