# Cx43 deficiency confers EMT-mediated tamoxifen resistance to breast cancer via c-Src/PI3K/Akt pathway: Erratum

**DOI:** 10.7150/ijbs.75891

**Published:** 2022-08-20

**Authors:** Deng-Pan Wu, Yan Zhou, Li-Xiang Hou, Xiao-Xiao Zhu, Wen Yi, Si-Man Yang, Tian-Yu Lin, Jin-Lan Huang, Bei Zhang, Xiao-Xing Yin

**Affiliations:** 1 Jiangsu Key Laboratory of New Drug Research and Clinical Pharmacy, Pharmacy School of Xuzhou Medical University, Xuzhou City, Jiangsu Province, 221004, P.R. China.; 2 Department of Pharmacology, Pharmacy School of Xuzhou Medical University, 221004, Xuzhou City, Jiangsu Province, P.R. China.; 3 Scientific research center of traditional Chinese medicine, Guangxi University of Chinese Medicine, Nanning City, Guangxi Zhuang Autonomous Region, P.R. China.; 4 Clinical Pharmacy, Jingjiang People's Hospital, 214500, Jingjiang City, Jiangsu Province, P.R. China.; 5 Department of gynaecology and obstetrics, Xuzhou Central Hospital, 221009, Xuzhou City, Jiangsu Province, P.R. China.

In our paper, there were some concerns of representative images of cell invasion and Western blot assays. During the preparation of representative images, cell invasion images of Control and TGF-β1 of Figure 4B, Cx43+TGF-β1 of Figure 5F and Cx43+18α-GA of Figure 7C were misplaced due to our carelessness in the selection of representative images. Western blot images of β-actin of Figure 3D, β-actin and Cx43 input of Figure 10A were misused due to errors in cropping and copy from the images of β-actin of Figure 3C, β-actin of Figure 9A and E-ca of Figure 9D, respectively. This is due to our mishandling when compiling images using PS software.

Given the experimental accuracy, we repeated the Western blot assay of Figure 3D and Co-IP assay in Figure 10A using the samples from the same cell lines as in the article. It should be noted that the updated results of Western blot and Co-IP assays are consistent with the conclusions presented in the article. We also traced back the original data of Figure 4B, 5F and 7C, the analysis of the data and the conclusion were not influenced since only representative images of these figures were changed.

All authors have agreed to the erratum. We feel regretful for not detecting these mistakes before publication and sincerely apologize for our mistakes and any inconvenience this might have caused. After correction, the figure legends of these figures were not influenced. The corrected figures are as follows.

## Figures and Tables

**Figure 3:**
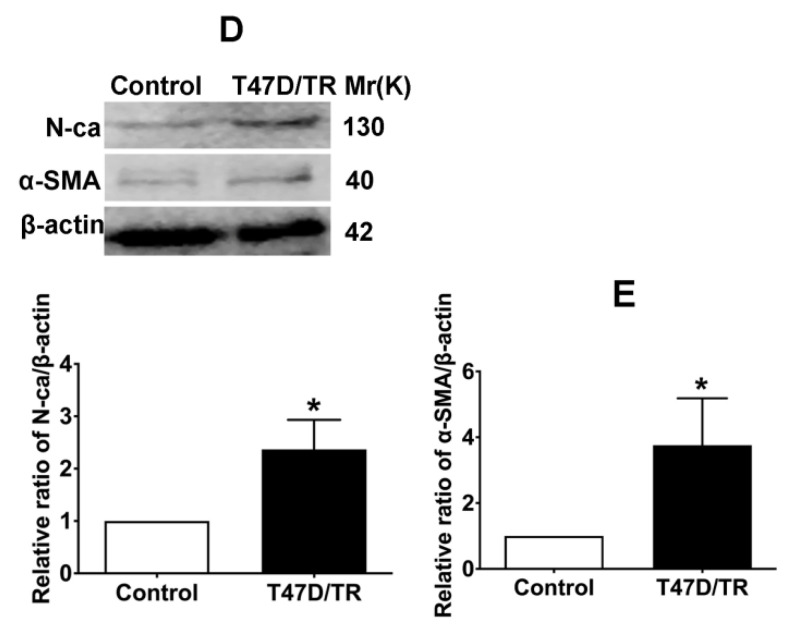
Corrected figure.

**Figure 4:**
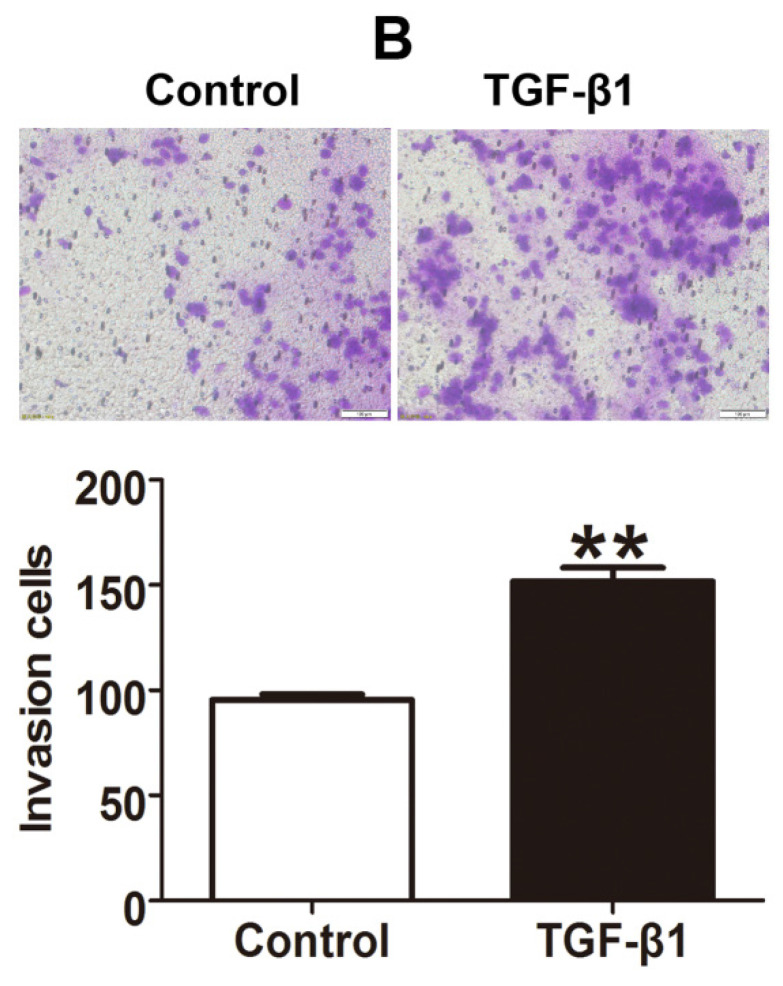
Corrected figure.

**Figure 5:**
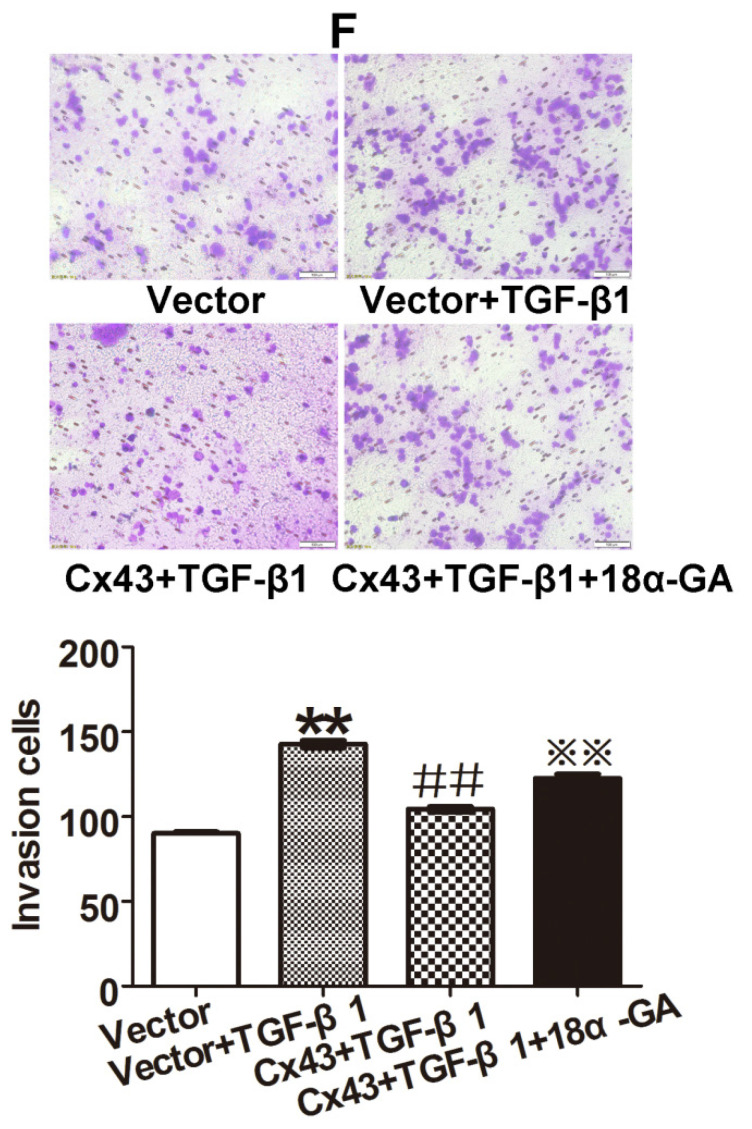
Corrected figure.

**Figure 7:**
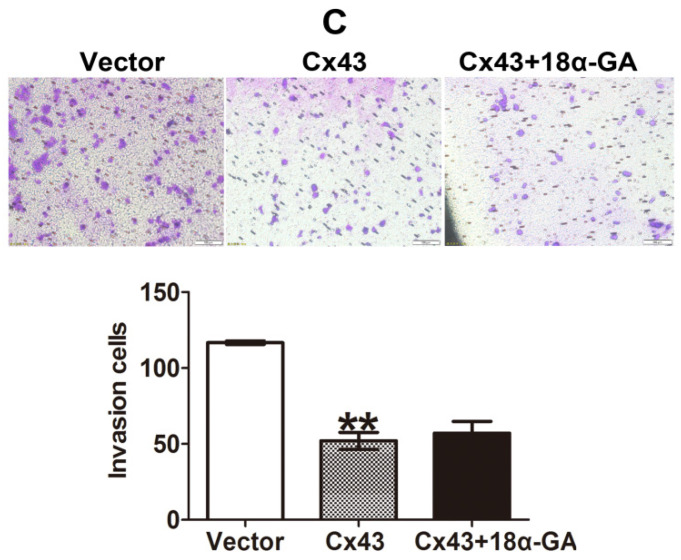
Corrected figure.

**Figure 10:**
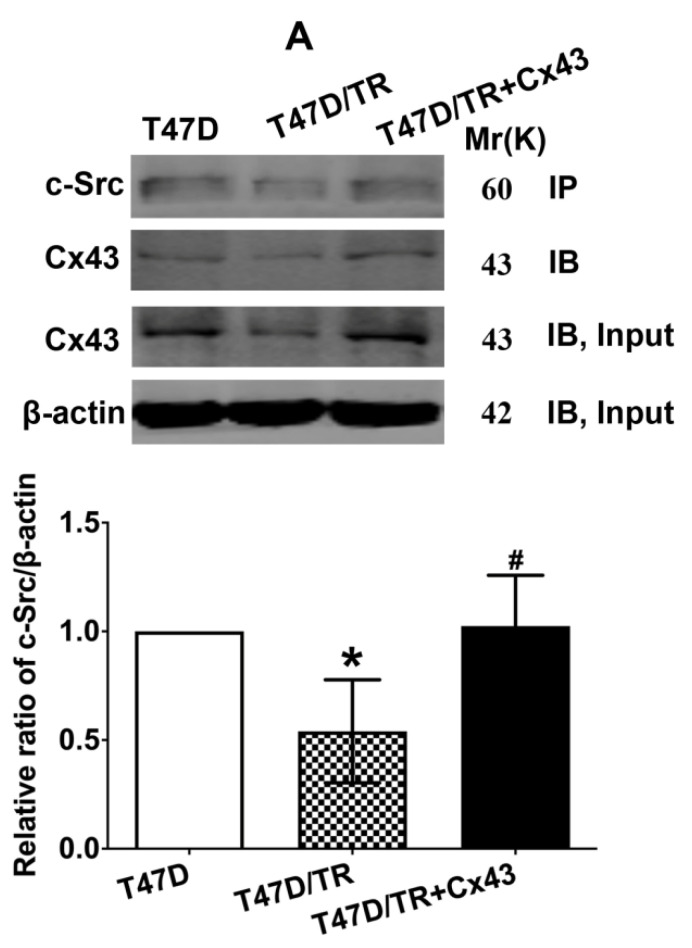
Corrected figure.

